# Hey1 promotes migration and invasion of melanoma cells via GRB2/PI3K/AKT signaling cascade

**DOI:** 10.7150/jca.60974

**Published:** 2021-10-11

**Authors:** Yihuan Pu, Mingxing Lei, Yangmei Chen, Yanran Huang, Lingzhao Zhang, Jiayi Chen, Yujie Zhang, Xinyi Shao, Lin Liu, Jin Chen

**Affiliations:** 1 Department of Dermatology, The First Affiliated Hospital of Chongqing Medical University, Chongqing 400016, China.; 2 111 Project Laboratory of Biomechanics and Tissue Repair, College of Bioengineering, Chongqing University, Chongqing 400044, China.; 3 Key Laboratory of Biorheological Science and Technology of the Ministry of Education, College of Bioengineering, Chongqing University, Chongqing 400044, China.; 4 Department of Orthopaedics, The First Affiliated Hospital of Chongqing Medical University, Chongqing 400016, China.

**Keywords:** Melanoma, Hey1, GRB2/PI3K/AKT signaling, EMT, Invasion

## Abstract

Increasing evidence indicates that Notch signaling regulates multiple intracellular biological processes in malignant melanoma. Whereas how Notch signaling is transduced to influence melanoma cell behaviors remains largely elusive. Here we show that the Notch signaling downstream target Hey1 promotes migration and invasion of melanoma cells via the GRB2/PI3K/AKT pathway. First, bioinformatics tools, immunohistochemistry, and Western blotting analysis showed that the expression of Hey1 is increased in melanoma. Then, both *in vivo* and *in vitro* experiments showed that Hey1 promotes the malignant behaviour of the melanoma cells. High-throughput RNA-sequencing analysis revealed that inhibition of Hey1 results in decreased GRB2 expression in melanoma cells. Last, functional experiments confirmed that Hey1 positively regulates GRB2/PI3K/AKT pathway to influence migration and invasion of melanoma cells. In summary, our results suggest that Hey1 promotes the invasion and metastasis of melanoma cells by regulating GRB2/PI3K/AKT pathway. Our study provides potential therapeutics in tumor biology.

## Introduction

Melanoma is a highly malignant and aggressive grade of tumor with a high incidence rate, rapid growth, rapid metastasis, and high degree of resistance to radiotherapy and chemotherapy, leading to poor prognosis and high mortality. The primary treatment methods for melanoma include surgical resection, chemoradiotherapy, and immunotherapy 1.

The main signaling pathways involved in the occurrence and development of melanoma include MAPK, p13k-AKT, TGF-β, Wnt, and Notch signaling 2. Previous studies have delineated a multifunctional role of Notch signaling, capable of controlling several aspects of melanoma pathogenesis. Notch signaling can synergistically interact with other signaling cascades such as Wnt, TGF-β, and ErbB signaling to influence melanoma cell behaviors 3. Hey1, one of the target genes of the Notch signaling pathway, encodes a transcription factor with a wide range of functions 4. Heisig et al. found that Hey1 modulates 507 genes involved in positive and negative transcription regulation, RNA splicing, chromatin assembly, protein transport, and organ development 5.

Hey1 plays important roles in regulating behaviors of various tumors. Tsuru et al. found that Hey1 expression was augmented in osteosarcoma, and Hey1 silencing inhibited the invasion and lung metastasis of this cancer 6. In colon cancer, enhanced Hey1 expression is linked to the invasion of peripheral nerves and blood vessels and lymph node metastasis 7. Park et al. found that Hey1 influenced the distant metastasis of breast cancer 8. In addition, Hey1 was reported to promote the metastasis of lung cancer 9 and malignant progression of bladder cancer 10. However, the molecular mechanism by which Hey1 regulates melanoma cell behaviors remains largely unknown. In the present study, we investigated the role of Hey1 in influencing human melanoma cells and elucidated that Hey1 promotes migration and invasion of melanoma cells via the GRB2/PI3K/AKT signaling pathway.

## Methods and materials

### Tissue samples and cell culture

Twenty-two normal skin tissues and twenty-seven melanoma tissues were collected from the Department of Dermatology, the First Affiliated Hospital of Chongqing Medical University. No patients had undergone radiotherapy or chemotherapy before the operation and all patients provided written informed consent before surgery. Utmost care was taken to ensure that the privacy rights of human participants were safeguarded. Specimen collection was approved by the clinical ethics committee of the First Affiliated Hospital of Chongqing Medical University and all procedures complied with the Declaration of Helsinki. The melanoma cell lines mel-888, mel-624, A375, and B16 were gifted by Dr. Tongchuan He (the University of Chicago Medical Center, Chicago, USA). The gll-19 cell line was donated by Dr. Qu Le (China Medical University, Shenyang, China). The human normal melanocyte cell line PIG1 was gifted by Dr. Chunying Li (Fourth Military Medical University, Shanxi, China). Melanoma cell lines were cultured in Dulbecco's Modified Eagle's Medium (HyClone, Logan, UT, USA) supplemented with 10% fetal bovine serum (Thermo Fisher Scientific, Waltham, MA, USA), 1% penicillin, and 1% streptomycin. PIG1 cells were cultured in 254 medium supplemented with human melanocyte growth supplement (Thermo Fisher Scientific), 5% fetal bovine serum (Thermo Fisher Scientific), 1% penicillin, and 1% streptomycin. All cells were cultured in a 5% CO2 incubator at 37 °C.

### Immunohistochemistry

Immunohistochemistry (IHC) analysis was performed using paraffin-embedded sections. An Immunohistochemistry sp-9000 Kit (Beijing Zhongshan Jinqiao Biotechnology Co., Ltd., Beijing, China) was used to stain and analyse all sections. The intensity of immunohistochemical staining and the average percentage of positive cells were evaluated.

### Gene Expression Profiling Interactive Analysis

The GEPIA online database (http://GEPIA.cancer-pku.cn/) was used to analyse the mRNA expression of Hey1 in melanoma. GEPIA samples were obtained from the Cancer Genome Atlas and Genotype Tissue Expression projects.

### Small interfering RNA transfection and adenovirus infection

The control small interfering RNA (siRNA) (s20c-0600) and Hey1 siRNA (sc-37913) were purchased from Biomics Biotech (Jiangsu, China). All siRNA transfection experiments were carried out according to the manufacturer's procedures using Lipofectamine RNAiMAX (Life Technologies, Carlsbad, CA, USA). Dr. Luo (The First Affiliated Hospital of Chongqing Medical University, Chongqing, China) gifted Hey1 overexpression (Ad-Hey1) and interference Hey1 recombinant adenovirus, as well as red and green fluorescent protein recombinant adenovirus as controls. Adenoviruses were transfected into A375 cells with polyethylene (Sigma Aldrich, St. Louis, MO, USA). Measurement of the fluorescence intensity 24 h later indicates that transfection was successful.

### Western blotting

The protein extract was separated by 10% sodium dodecyl sulphate-polyacrylamide gel electrophoresis and then transferred to the polyvinylidene fluoride (PVDF) membranes. The membrane was placed in a blocking solution (Shanghai Biyun Tian Biotechnology Co., Ltd., Shanghai, China) for 2 h at 37 °C. Next, the membrane was incubated overnight with the target primary antibody Hey1 (1:1,000; Abcam, Cambridge, MA, USA), E-cadherin (1:5,000; Abcam), GRB2 (1:1,000; Abcam), N-cadherin (1:1,000; Abcam), matrix metalloproteinase-2 (MMP-2, 1:1,000; Abcam), MMP-9 (1:1,000; Cell signaling Technology, Danvers, MA, USA), MMP-7 (1:1,000; Cell signaling Technology), Snail (1:1,000; Cell signaling Technology) vimentin (1:1,000; Cell signaling Technology), PI3K (1:1000; Cell signaling Technology), Phosphorylated PI3K (1:1000; Affinity Biosciences, Jiangsu, China), AKT (1:1000; Cell signaling Technology), Phosphorylated AKT (ser308, 1:1000; Cell signaling Technology), and Phosphorylated P21 (1:1000; ImmunoWay Biotechnology, Plano, TX, USA), then washed with Tris-buffered saline containing Tween 20 three times, followed by incubation with an appropriate secondary antibody (1:5000; Beijing Zhongshan Jinqiao Biotechnology Co., Ltd., Beijing, China) at 37 °C for 1 h. Protein bands were visualised using the SuperSignal West Pico chemiluminescence substrate Kit (EMD Millipore, Billerica, MA, USA). Each experiment was repeated thrice.

### Cell proliferation assay

Cell viability was detected by CCK8 assay. 3,000 cells were seeded into each well of 96-well plates. After 0, 24, 48, 72, or 96 h of culture, CCK8 solution (Cell Counting kit-8, Dojindo, Kumamoto, Japan) was added to each well and the cells were further incubated at 37 °C for 1 h. The optical density (OD) was measured at 450 nm with a microplate reader.

### Colony-formation assay

Log-phase cells were seeded into 6 well-plates at a density of 800 cells/well and incubated until clones were observed. The cells were then fixed with 4% paraformaldehyde and stained with 0.1% crystal violet. Visible colonies were counted.

### Migration, invasion, and wound-healing analysis

A Transwell chamber (24-well Transwell chamber, 8 μm; Corning, Inc., Corning, New York, USA) was used for migration and invasion analysis. At 48 h after transfection, the cells were re-suspended in the serum-free medium, and then cells at a density of 50,000/200 μL were inoculated into the upper chamber. High-glucose medium containing 10% fetal bovine serum was added to the lower chamber. The cells were cultured for 24 h and stained with 0.1% crystal violet. The Transwell invasion assay was performed similarly to the migration assay, but the bottom of the Transwell chamber was coated with Matrigel at a dilution of 1:10. In wound-healing analysis, the cells were seeded into a 6-well plate. When the transfected cells grew to 90%, a 200 μL pipette was used to scratch the wound, and the cells were incubated for 24 h. Specific scratch areas were photographed at 0 and 24 h. Cell migration was evaluated by mobility: (original scratch width - new scratch width)/original scratch width × 100%. Each experiment was repeated thrice.

### High-throughput sequencing analysis

Total RNA was isolated using the RNeasy mini kit (Qiagen, Germany). Paired-end libraries were synthesized by using the TruSeq™ RNA Sample Preparation Kit (Illumina, USA) following Sample Preparation Guide. The cleaved RNA fragments are copied into first-strand cDNA using reverse transcriptase and random primers. This is followed by second-strand cDNA synthesis using DNA Polymerase I and RNase H. These cDNA fragments then go through an end repair process, the addition of a single 'A' base, followed by ligation of the adapters. The products are next purified and enriched with PCR to create the final cDNA library. Purified libraries were quantified by the Qubit®2.0 Fluorometer (Life Technologies, USA) and validated by the Agilent 2100 bioanalyzer (Agilent Technologies, USA) to confirm the insert size and calculate the mole concentration. Cluster was generated by cBot with the library diluted to 10 pM and then was sequenced on the Illumina NovaSeq 6000 (Illumina, USA).

### Immunofluorescence

Immunofluorescence (IF) analysis was performed using paraffin-embedded sections. Paraffin sections were dehydrated in sequential order. Auto-fluorescence was quenched with BSA. The samples were incubated with primary antibodies and secondary antibodies (Alexa 488 and FITC, Servicebio, Wuhan, China) at a dilution of 1:5000, and then mounted by DAPI staining reagent (Servicebio). Samples were visualized under a Pannoramic Scanner system (Pannoramic DESK, P-MIDI, P250, 3D HISTECH, Hungary).

### Xenotransplantation in nude mice

Approximately 5 × 10^6^ adenovirus-transfected A375 cells were suspended in 100 μL serum-free medium and then injected into nude mice. Female nude mice were employed in the study. The tumor size was recorded with a Vernier caliper every week, and the tumor volume was calculated using the following formula: length × width 2 × 0.5. Four weeks later, the mice were sacrificed by cervical disc dislocation. Tumour tissue and lung tissue were collected and embedded in paraffin for IHC and hematoxylin and eosin analysis. All procedures related to animal handling, care, and treatment were complied with the ARRIVE guidelines and were carried out in accordance with the National Institutes of Health guide for the care and use of laboratory animals. The scheme was also approved by the Animal Ethics Committee of the First Affiliated Hospital of Chongqing Medical University.

### Statistical analysis

Data are expressed as the mean ± standard deviation, and all values were analysed using GraphPad Prism (Prism 8.0.2; GraphPad Software, Inc., La Jolla, CA, USA). The student's t-test was used to evaluate the differences between the experimental group and control group. P < 0.05 was considered to indicate statistically significant results.

## Results

### Hey1 expression was increased in human melanoma tissues and melanoma cells

**Figure 1:**
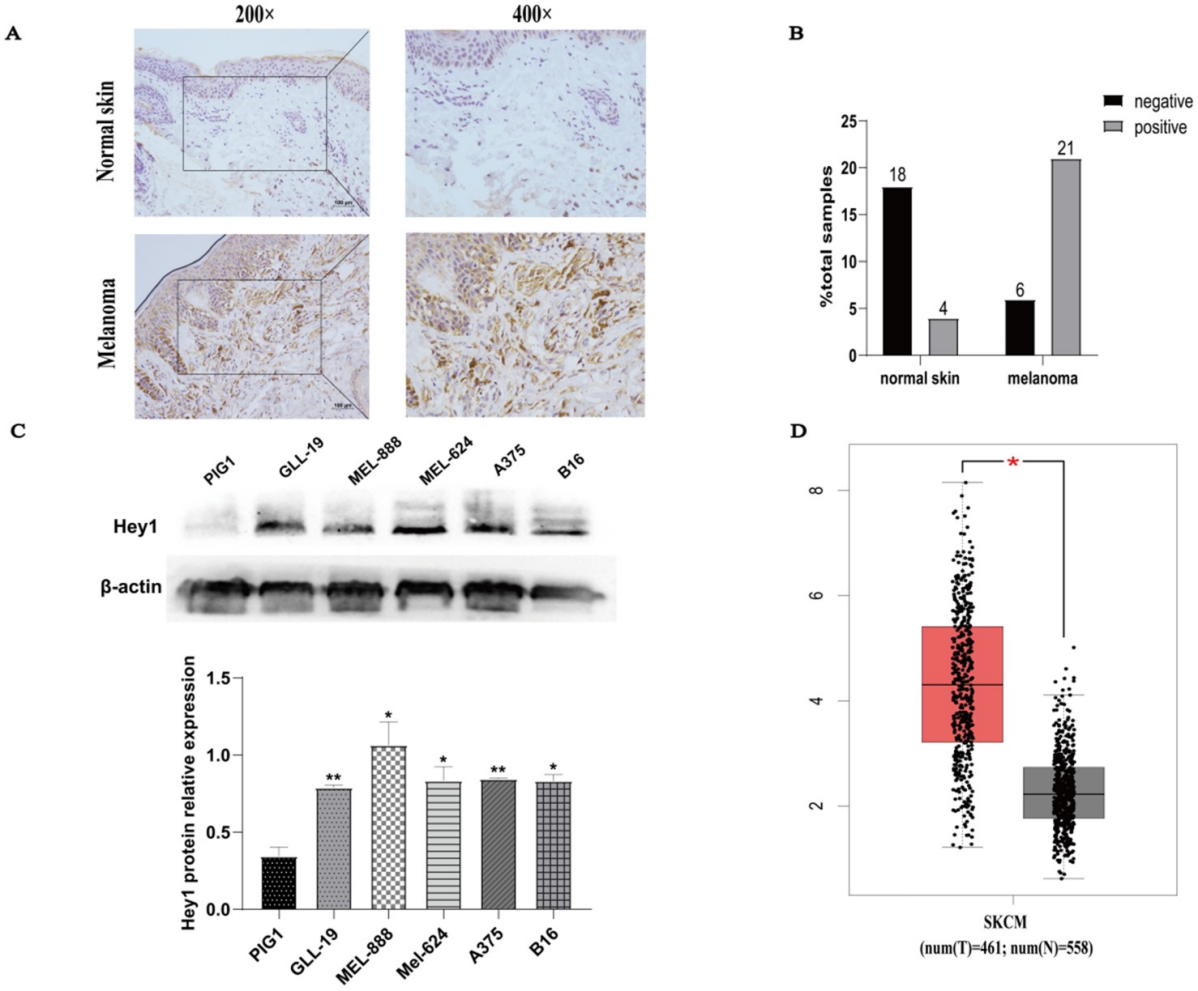
** Hey1 expression is upregulated in melanoma tissues and melanoma cell lines. (A)** Representative immunohistochemical staining of Hey1 in normal skin (n=22) and melanoma tissue (n=27) paraffin sections. **(B)** The percentage of Hey1-negative and -positive staining scores in normal skin and melanoma tissues. **(C)** Hey1 expression was measured by Western blotting analysis in human epidermal melanocytes PIG1 cell line and melanoma cell lines. Data are shown as mean±SD.*P<0.05, **P<0.01, ***P<0.001. **(D)** Hey1 expression in normal skin (n=558) and skin cutaneous melanoma (n=461) in the GEPIA database. **Abbreviations:** GEPIA, Gene Expression Profiling Interactive Analysis; Hey1, Hes related family bHLH transcription factor with YRPW motif 1.

Hey1 expression was first examined in normal human skin and melanoma tissues by IHC. The Hey1 expression level was higher in melanoma tissue than in normal skin tissue (Fig. 1A). Samples were scored on the basis of immunoreactivity scores, negative (1-4) and positive (5-12) 11. The positivity rate of Hey1 was 18% (4/22) in normal skin and 77% (21/27) in melanoma tissue (Fig. 1B). The expression of Hey1 protein was significantly higher in melanoma cells (gll-19, mel-888, mel-624, A375, and B16) than that in the PIG1 cells (Fig. 1C). The Hey1 mRNA expression level was significantly upregulated in melanoma tissues compared to the corresponding expression level in normal skin tissues (Fig. 1D). As the A375 cell line is widely used for melanoma investigations, it was also employed herein to examine the role of Hey1 in melanoma.

### Hey1 promoted the invasion, migration, and EMT process of melanoma cells

**Figure 2:**
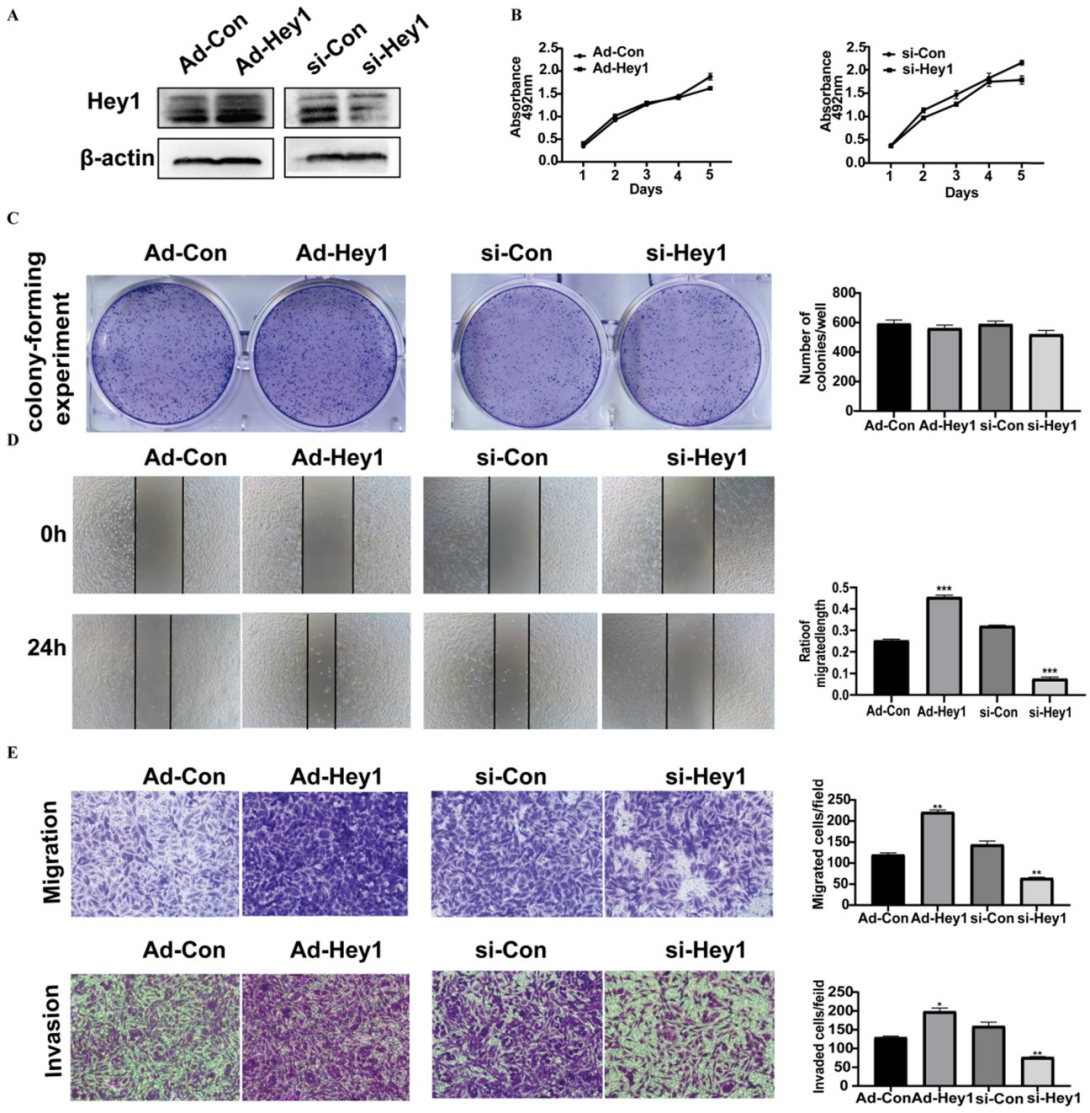
** Hey1 does not alter cell proliferation, but promotes the invasion and migration of melanoma cells. (A)** Western blotting analysis of the Hey1 expression levels in Hey1 siRNA-treated A375 cells and in Ad-Hey1-infected A375 cells. **(B)** Cell viability was measured by the CCK8 assay in A375 cells. **(C)** Images of colony formation and depiction of the colony formation rates. **(D)** The representative images (magnification ×200) and percentages of cell migration. **(E)** The representative images (magnification ×200) and the number of migrated and invaded cells. Data are shown as mean ± SD. *P<0.05, **P<0.01, ***P<0. 001. Data represent three independent experiments. **Abbreviations:** Con: control; si: small interfering RNA; Ad: recombinant adenoviruses; CCK8, 2 - (2-methoxy-4-nitrophenyl) - 3 - (4-nitrophenyl) - 5 - (2,4-disulfonic acid benzene) - 2H tetrazole monosodium salt; EMT, epithelial-mesenchymal transition.

Hey1 protein expression was first examined in adenovirus- and siRNA-treated A375 cells by Western blotting. Its expression level was significantly elevated in the overexpression group and decreased in the interference group, compared to the expression level in the corresponding control group (Fig. 2A). Then, the CCK8 assay (Fig. 2B) and Colony-forming assay (Fig. 2C) were used to detect the impact of Hey1 on the proliferation of A375 cells. The results showed that Hey1 did not significantly affect the proliferation of A375 cells. However, wound-healing analysis (Fig. 2D) and Transwell invasion assay (Fig. 2E) results showed that the migration and invasion abilities were significantly enhanced in the Hey1 overexpression group and inhibited in the Hey1 interference group, when compared to the corresponding control groups.

**Figure 3:**
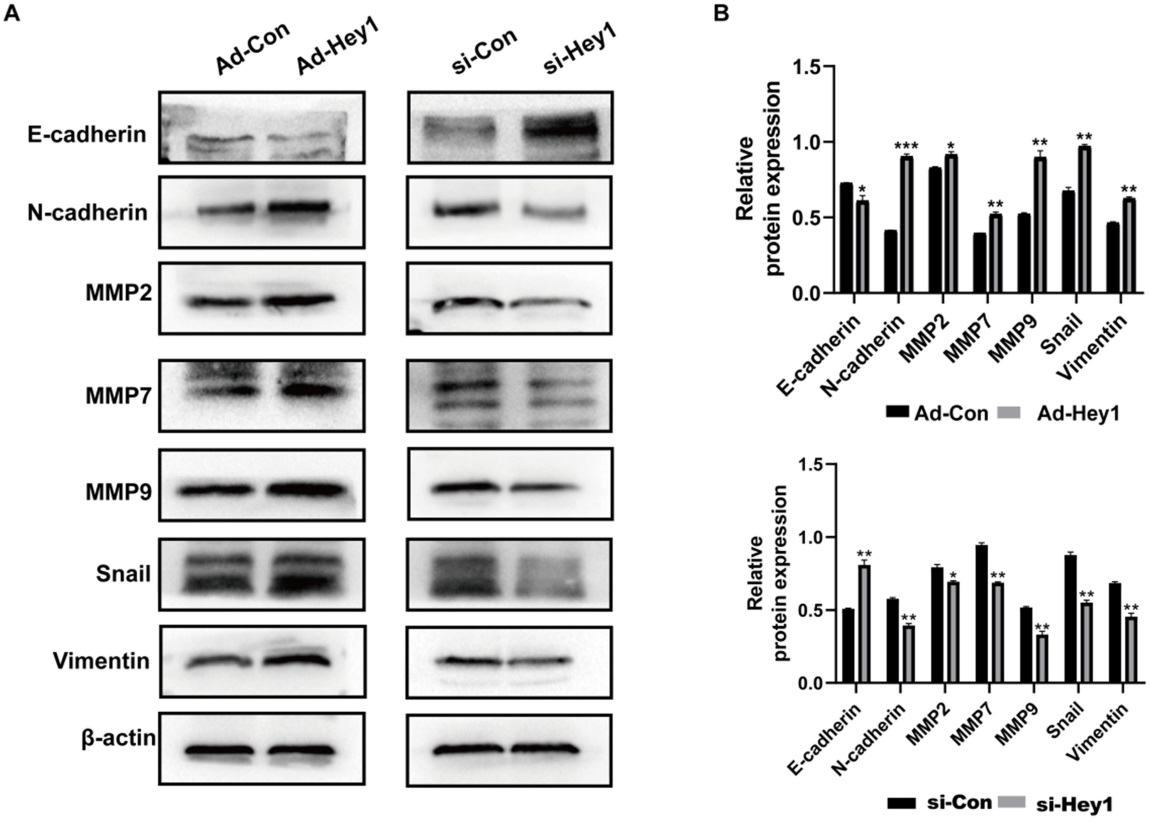
** Hey1 promotes EMT and MMPs protein expression of melanoma cells. (A)** Western blotting analysis of protein expression levels of E-cadherin, N-cadherin, MMP-9, MMP-7, MMP-2, vimentin, and Snail. **(B)** Relative expression of each protein to β-actin in A375 cells. Data are shown as mean ± SD. *P<0.05, **P<0.01, ***P<0.001. Data represent three independent experiments. **Abbreviations:** EMT, epithelial-mesenchymal transition; MMP: matrix metalloproteinase.

Epithelial-mesenchymal transition is an important step in early tumor metastasis 12. The protein matrix metalloproteinases (MMPs) can degrade the extracellular matrix to promote tumor invasion 13. Previous studies have shown that Hey1 can promote the EMT process and the expression of matrix metalloproteinases expression profile of tumor cells 14-16. Here, Western blotting was used to detect the impact of Hey1 on the expression of EMT-related proteins in A375 cells. The results showed that the expression of E-cadherin was significantly increased and the expression of N-cadherin, MMP-2, MMP7, MMP-9, Vimentin, and Snail was significantly decreased in the Hey1 interference group, compared to that of the controls. On the contrary, the expression of E-cadherin was significantly decreased and the expression of N-cadherin, MMP-2, MMP7, MMP-9, Vimentin, and Snail was significantly increased in the Hey1 overexpression group, compared to that of the controls (Fig. 3A-B).

### Hey1 promoted the growth and lung metastasis of melanoma cells in nude mice

**Figure 4:**
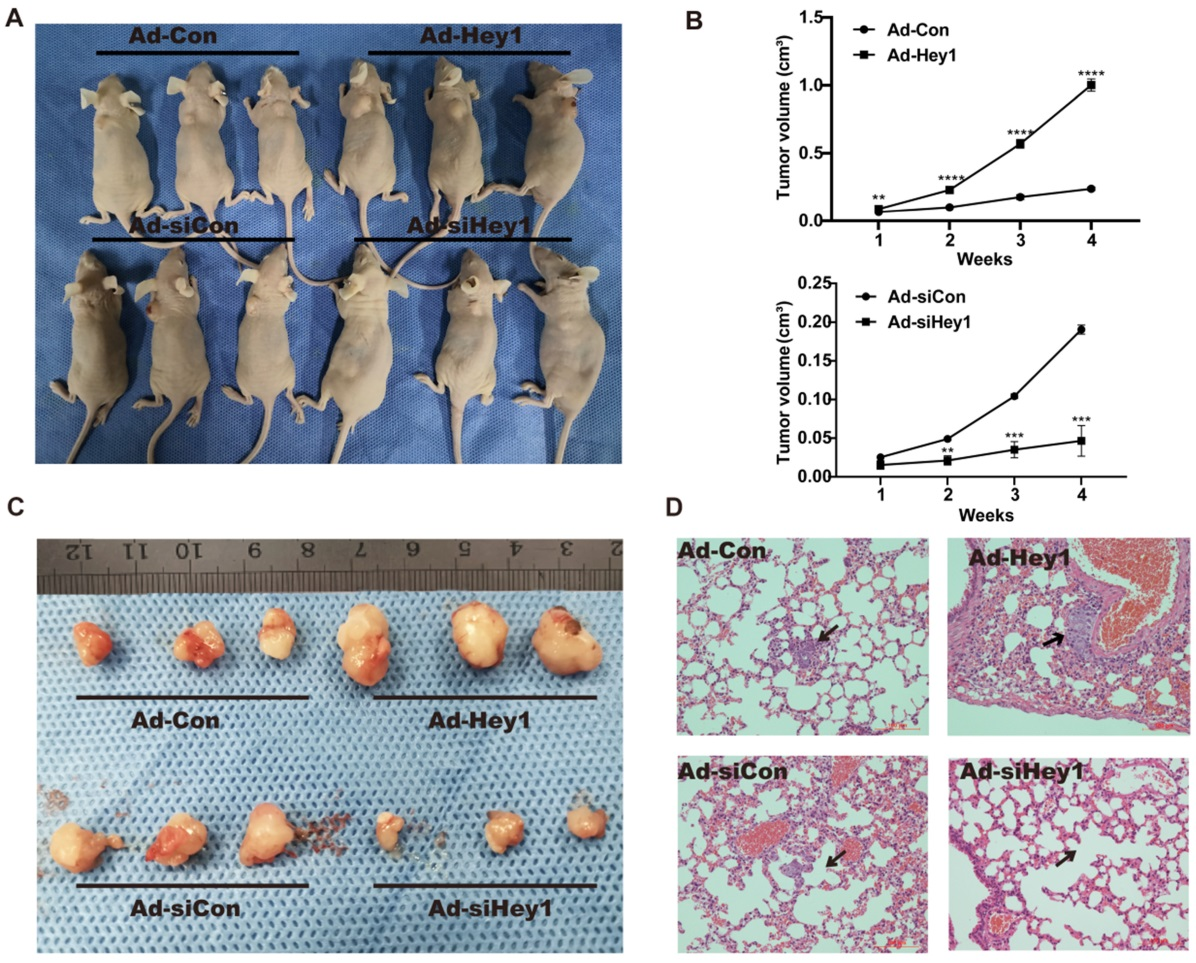
** Hey1 interference suppresses and Hey1 overexpression promotes tumor growth and lung metastases of A375 cells *in vivo*. (A)** These graphs show the tumor xenografts 4 weeks after ectopic-subcutaneous implantation in nude mice. **(B)** Tumour growth curve. **(C)** Representative images of primary implanted tumor mass of mice sacrificed at 4 weeks. **(D)** Representative hematoxylin and eosin images of lung specimens. Black arrow indicates metastatic lung nodule. Data are shown as mean ± SD. *P<0.05, **P<0.01.

A xenograft animal model was used to study the effect of Hey1 on the growth and metastasis of melanoma cells *in vivo*. Tumour volume was measured once per week. Compared with the tumor volume in the control group, the tumor volume was significantly reduced in the Hey1 interference group but was significantly increased in the Hey1 overexpression group (Fig. 4A-C). Subsequently, we collected lung tissues from tumorigenic mice for hematoxylin and eosin staining. Compared with the control group, there were smaller and fewer metastatic tumor islands in the Hey1 interference group and larger and more metastatic tumor islands in the Hey1 overexpression group (Fig. 4D). These results demonstrated that Hey1 promotes the metastasis and invasion of melanoma cells *in vivo*.

### Hey1 interference negatively regulated GRB2 expression and inhibited the PI3K/AKT signaling pathway in A375 cells

**Figure 5:**
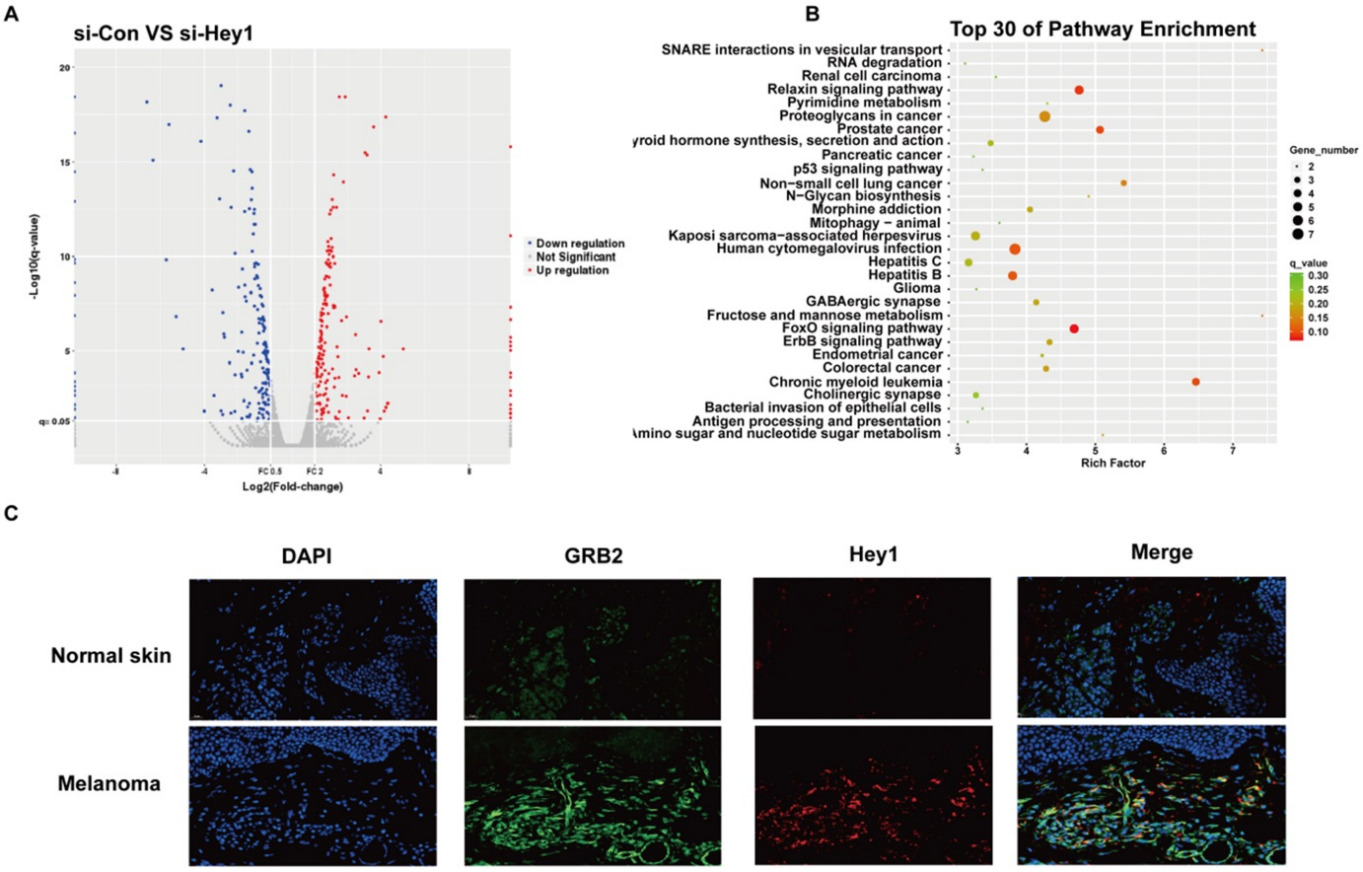
** High-throughput sequencing results of A375 interfering with Hey1 and immunofluorescence results of melanoma tissues. (A)** Volcano plot shows the gene expression in si-Hey1 A375 cells and control cells, and 232 differentially expressed genes (>2-fold change) were identified. **(B)** Top 30 genes identified in KEGG pathway enrichment. (C) The Hey1 and GRB2 protein expression levels were detected in melanoma tissues by immunofluorescence. **Abbreviations:** si: small interfering RNA; KEGG: Kyoto Encyclopedia of Genes and Genomes.

High-throughput sequencing was performed to characterize the effector molecules regulated by Hey1. A total of 232 coding genes were identified and visualised in a scatter plot (Fig. 5A). Kyoto Encyclopedia of Genes and Genomes (KEGG) analysis was performed on differential gene expression and the top30 were displayed (Fig. 5B). Since p53 and FoxO signaling pathways were largely studied in melanoma cells 17,18, we selected the ErbB signaling pathway for further verification. Among ErbB signaling pathway genes (Supplementary 1), we observed that the mRNA level of the adaptor protein GRB2 was significantly decreased after inhibition of Hey1 in melanoma cells, compared to that of the control. Then, we performed double immunofluorescence labeling of them in melanoma tissues. Intriguingly, in addition to Hey1, we observed GRB2 expression was also significantly increased in the melanoma tissues compared to that of the normal skin tissues (Fig. 5C). These results indicate that Hey1 may positively regulate GRB2 expression in melanoma tissue.

**Figure 6:**
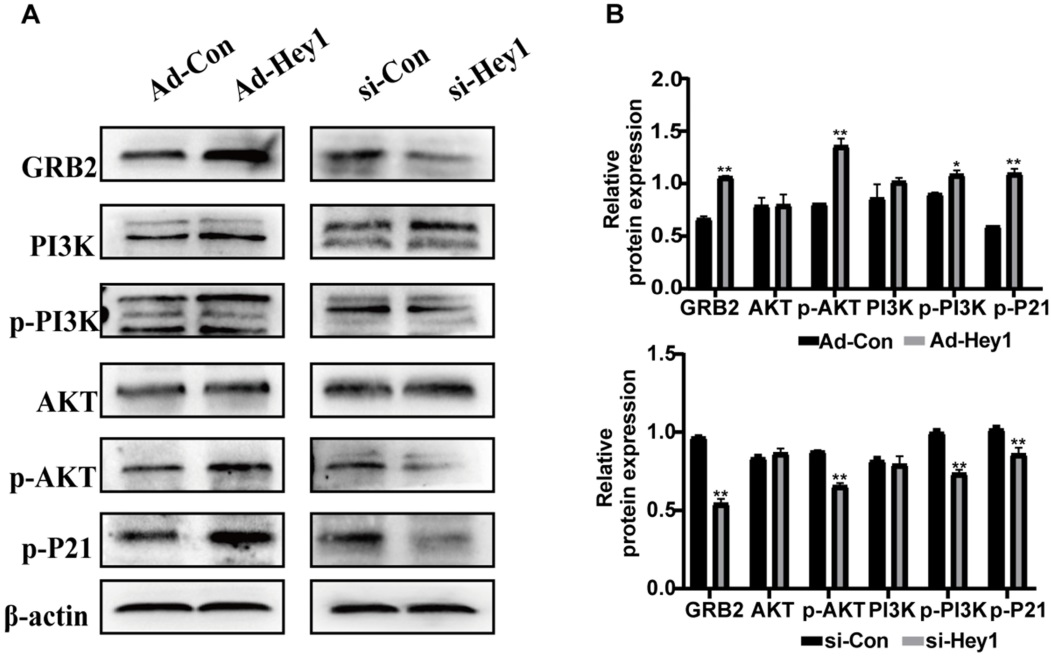
** Hey1 interference negatively regulates GRB2 expression and attenuates the GRB2/PI3K/Akt signaling pathway in A375 cells.** The protein expression levels of GRB2, PI3K, p-PI3K, AKT, p-AKT, and p-P21, as well as the relative expression of each protein to β-actin, compared with the corresponding control expression levels. **(A)** Akt and PI3K levels were not significantly changed. GRB2, p-PI3k, p-AKT (ser308), and p-P21 levels were significantly decreased in the A375-interference group. The corresponding expression levels noted in the overexpression group showed an opposite pattern. Data are shown as mean ± SD. *P<0.05, **P<0.01, ***P<0.001. Data represent three independent experiments.

**Figure 7:**
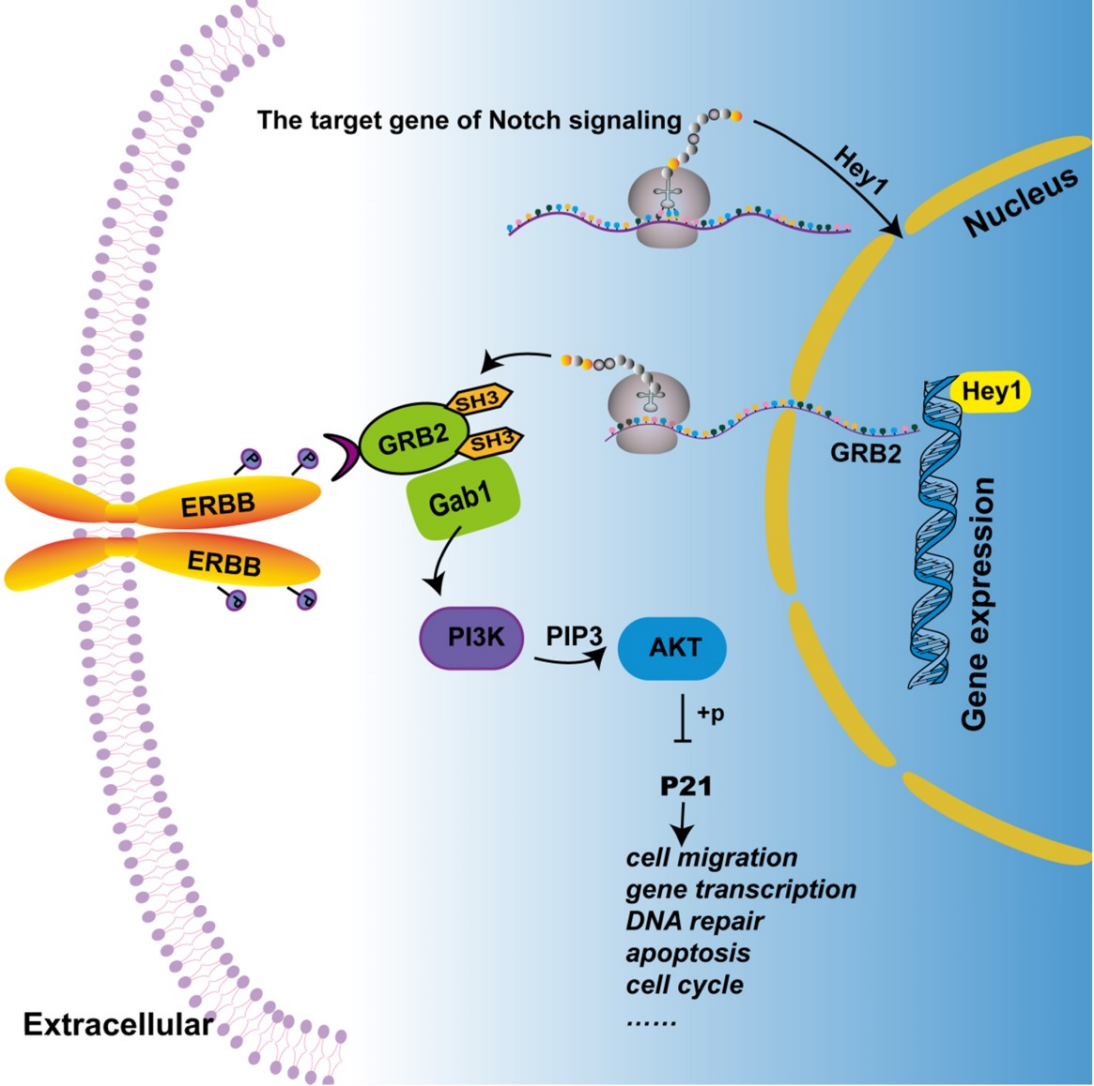
** Hey1 regulates the PI3K/Akt signaling pathway by regulating GRB2 expression in melanoma cells.** Combined with the previous and the present study, we depict that Hey1 promotes the expression of GRB2. GRB2 recruits and activates phosphatidylinositol-3-kinase (PI3K) to produce second messenger PIP3 by binding with Gab1 (GRB2 associated binding protein). PIP3 then phosphorylates and activates AKT, which acts on P21, thus affecting the biological process of the tumor.

Previous study showed that GRB2 can activate PI3K/AKT signaling in colorectal cancer cells 19 and the PI3K/AKT pathway regulates cell differentiation, migration, and invasion 20. In the present study, KEGG analysis showed that among the PI3K/AKT signaling, and the phosphorylation level of the P21 target gene was significantly decreased in the Hey1 inhibition group, compared to that of the control group. Thus, Western blotting was used to detect the expression of key proteins in the GRB2/PI3K/AKT signaling cascade. The results showed that the total AKT and PI3K protein expression did not significantly change, However, GRB2, p-PI3K, p-AKT (ser308), and p-P21 protein expression was significantly decreased in the Hey1 inhibition group, compared to that of the controls (Fig. 6A-B). In contrast, the expression of these proteins was significantly increased in the Hey1 overexpression group, compared to that of the control group. These results indicated that Hey1 regulates melanoma cell behavior through GRB2/PI3K/AKT signaling pathway (Fig. 7).

## Discussion

Significant progress has been made in treating melanoma in the past decades. However, melanoma remains a lethal cancer type, especially in the late stages of diagnosis. Thus, investigation of the progression of melanoma is positioned as a very important issue in tumor biology. In the present study, we investigated the crucial role of Hey1, a target gene of Notch signaling, on the biological function of melanoma and its molecular mechanisms. We found that Hey1 mediates GRB2 to regulate PI3K/AKT signaling in melanoma cells.

Our results showed that Hey1 is upregulated in melanoma tissues. Hey1 interference inhibited the migration and invasion of melanoma cells, while Hey1 overexpression exerted the opposite effects on melanoma cell behaviour and signaling transduction. We found that GRB2 and Hey1 have a similar expression trends. GRB2 is an adaptor protein and an important link in the receptor tyrosine kinase signaling cascade 21, providing a key association between cell-surface growth factor receptors and PI3K/AKT signal transduction 22. Prior studies have shown that an imbalance in the GRB2 level in tumors can influence the invasion and metastasis of tumor cells in malignancies, such as in liver cancer and lung cancer 23,24. Relatively, few studies have investigated the role of GRB2 in melanoma. One study showed that the cationic KT2 peptide downregulates GRB2 expression to inhibit the migration and invasion of human melanoma cells 25. Another study indicated that phycocyanin hinders the proliferation of melanoma cells by downregulating GRB2/ERK signaling transduction 26. However, we propose that Hey1 may augment the expression of GRB2 to promote the invasion and metastasis of melanoma cells. Notably, differential gene screening of human melanocytes of skins showed that GRB2 expression was downregulated during the differentiation and proliferation of human melanocytes 27, indicating that GRB2 is also linked to the malignant transformation of melanocytes.

The PI3K/AKT pathway is typically activated in melanoma 28. These activation signals include receptor tyrosine kinases, and GRB2 provides a key link between PTKs and PI3K/AKT. When PI3K binds to GRB2 and is recruited to the cell membrane for activation by phosphorylation, AKT is then also recruited to the cell membrane and phosphorylated. Phosphorylated AKT acts on its target genes to regulate a variety of cellular processes 29. P21 is a target gene of PI3K/AKT signaling pathway, to regulate various cellular processes including DNA repair, apoptosis, differentiation, cytoskeleton dynamics, cell migration, gene transcription, and reprogramming induced pluripotent stem cells. Interestingly, P21 not only plays an anti-cancer role but also generates a carcinogenic effect. This dual behaviour depends on its subcellular localisation, interaction partner, and phosphorylation state 30, 31. We found that decreased phosphorylation of PI3K and AKT inhibited phosphorylation of P21, implying that Hey1 indirectly regulates the PI3K/AKT/P21 signaling network via GRB2.

Furthermore, previous studies revealed signaling crosstalks between the Notch and ERBB signaling pathways, which are activated simultaneously in melanoma to exert synergistic effects 32. A review mentioned that Notch and ERBB signaling components (i.e., Notch1, Hey1, and ERBB3) are re-expressed in melanoma in a correlative manner, and proposed that the tumorigenesis and development of melanoma require two cascade functions 33. Our study confirmed part of the hypothesis that Hey1, as the main component of Notch signaling, participates in the ERBB signaling pathway by activating the GRB2/PI3K/AKT signaling cascade.

In summary, we showed that Hey1 regulates the PI3K/AKT signaling pathway via GRB2 mediation. Hey1 promotes the EMT process, tumor matrix metalloproteinases expression, as well as the migration and invasion of melanoma cells. Nevertheless, whether Hey1 binds on the GRB2 promoter to regulate its expression remains further investigation. In conclusion, our study provides compelling evidence that Hey1 promotes melanoma cell migration and invasion through GRB2/PI3K/AKT signaling cascade.
